# A novel mutation in *PRKAR1A* gene in a patient with Carney complex presenting with pituitary macroadenoma, acromegaly, Cushing's syndrome and recurrent atrial myxoma

**DOI:** 10.20945/2359-3997000000369

**Published:** 2021-04-29

**Authors:** Ali A. Ghazi, Mohammad Hossein Mandegar, Mohammad Abazari, Neda Behzadnia, Taraneh Sadeghian, Siamak Shariat Torbaghan, Alireza Amirbaigloo

**Affiliations:** 1 Shahid Beheshti University of Medical Sciences Research Institute for Endocrine Sciences Endocrine Research Center Tehran Iran Endocrine Research Center, Research Institute for Endocrine Sciences (RIES), Shahid Beheshti University of Medical Sciences, Tehran, Iran; 2 Tehran University of Medical Sciences Section of Cardiothoracic Surgery Tehran Iran Section of Cardiothoracic Surgery, Tehran University of Medical Sciences, Tehran, Iran; 3 Shahid Beheshti University of Medical Sciences Section of Cardiovascular Disorders Tehran Iran Section of Cardiovascular Disorders, Shahid Beheshti University of Medical Sciences, Tehran, Iran; 4 Shahid Beheshti University of Medical Sciences National Institute of Tuberculosis and Lung Disease Lung Transplantation Research Center Tehran Iran Lung Transplantation Research Center, National Institute of Tuberculosis and Lung Disease (NRILTD), Shahid Beheshti University of Medical Sciences, Tehran, Iran; 5 Azad University of Medical Sciences Section of Dermatology Tehran Iran Section of Dermatology, Azad University of Medical Sciences; Consultant Dermatologist at Kasra General Hospital, Tehran, Iran; 6 Tehran University of Medical Sciences Imam Khomeini Hospital Department of Pathology Tehran Iran Department of Pathology, Imam Khomeini Hospital, Tehran University of Medical Sciences, Tehran, Iran; 7 Karaj Iran Endocrinologist, Practicing at Private Practice, Karaj, Iran

## Abstract

Carney complex (CNC) is a rare syndrome of multiple endocrine and non-endocrine tumors. In this paper we present a 23-year-old Iranian woman with CNC who harbored a novel mutation (c.642dupT) in *PRKAR1A* gene. This patient presented with pituitary macroadenoma, acromegaly, recurrent atrial myxoma, Cushing's syndrome secondary to primary pigmented nodular adrenocortical disease and pigmented schwanoma of the skin. *PRKAR1A* gene was PCR amplified using genomic DNA and analyzed for sequence variants which revealed the novel mutation resulting in substitution of amino acid cysteine instead of the naturally occurring valine in the peptide chain and a premature stop codon at position 18 (V215CfsX18). This change leads to development of tumors in different organs due to lack of tumor suppressive activity secondary to failure of synthesis of the related protein.

## INTRODUCTION

Carney complex (CNC) is a rare form of multiple endocrine and non-endocrine tumor syndrome that affects both males and females. Major clinical manifestations are pigmented mucocutaneous nevi, acromegaly secondary to pituitary somatotroph adenoma, Cushing's syndrome (CS) secondary to primary pigmented nodular adrenocortical disease (PPNAD), atrial myxoma and tumors of other organs such as thyroid, testes and ovaries. The disease is rarely encountered and less than 1,000 cases have been so far reported (1,2).

CNC is genetically heterogenous. In approximately 70% of cases the syndrome results from mutations in *PRKAR1A* gene which codes for regulatory subunit type 1 alpha of protein kinase A. The disease is transmitted through autosomal dominant pattern in 2/3 of cases. De novo mutations seem to be the cause of disease in the remaining 1/3. More than 125 pathogenic mutations have so far been reported in patients with CNC (3,4).

Majority of cases have been reported from North America and also European countries and there are limited information on clinical and genetic characteristics of CNC in Middle East region. In this paper, we present an Iranian patient with CNC who harbored a novel mutation (c.642dupT) in *PRKAR1A* gene and presented with acromegaly, recurrent atrial myxoma and CS.

## CLINICAL REPORT

A 23-year-old Iranian woman was admitted to the ward of thoracic surgery at Kasra General Hospital, Tehran, Iran because of recurrence of cardiac myxoma. She was well until 4 years ago when symptoms began gradually with palpitation, dyspnea and exercise intolerance. Cardiovascular evaluation at that time revealed a 7 x 5 x 3 cm left atrial myxoma that was operated at another hospital. After surgery she gradually developed edema of the face, enlargement of hands and feet and recurrent headaches. Two years ago, evaluations by an endocrinologist revealed that serum growth hormone was 112 mIU/L (normal values 0-55) and was not suppressed by oral glucose tolerance test. Pituitary MRI revealed a pituitary macroadenoma and one year ago she underwent transsphenoidal pituitary surgery in 2 sessions at another hospital. Histopathologic evaluation revealed a pituitary adenoma. The patient felt well until 6 months ago when she developed palpitation and exertional dyspnea. She also complained of facial puffiness and edema of hands and feet. Hormonal evaluation showed high GH [112 mIU/L (normal 0-50)] and high IGF1 799 ng/mL [(normal values for age 115-340)]. Sandostatin LAR 20 mg every month was started for the patient. She had regular menstrual cycles and personal history was otherwise negative. Family history was also negative for similar disorder.

**Figure 1:**
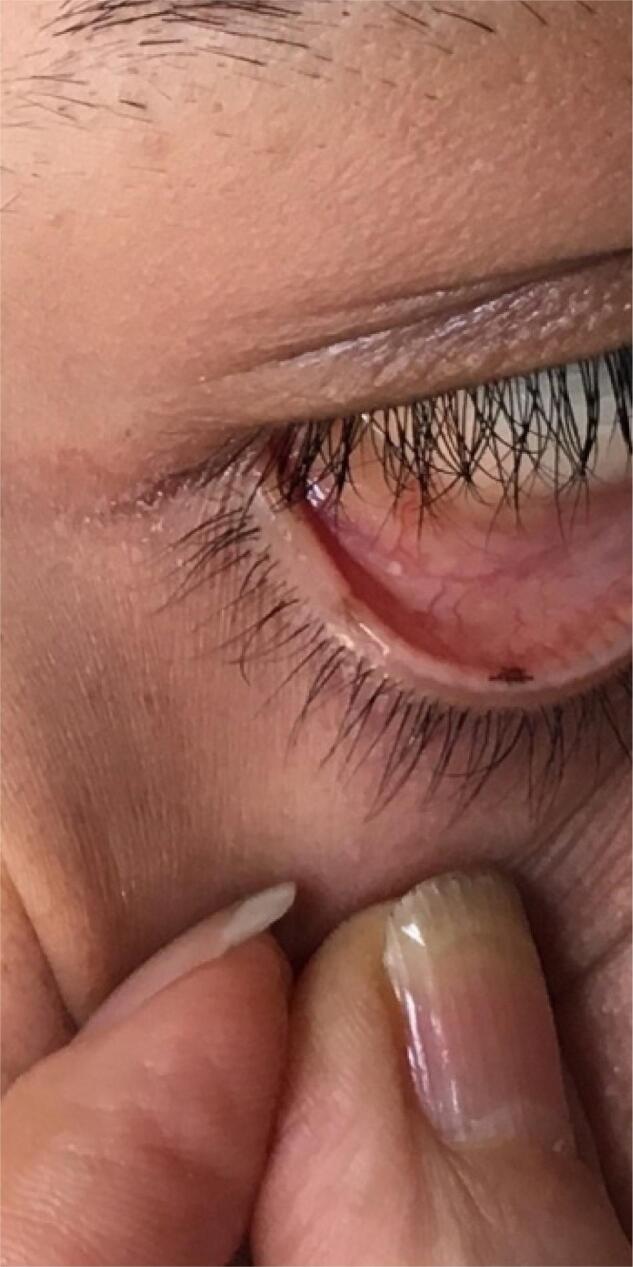
Pigmented macule in palpebral conjunctiva.

**Figure 2:**
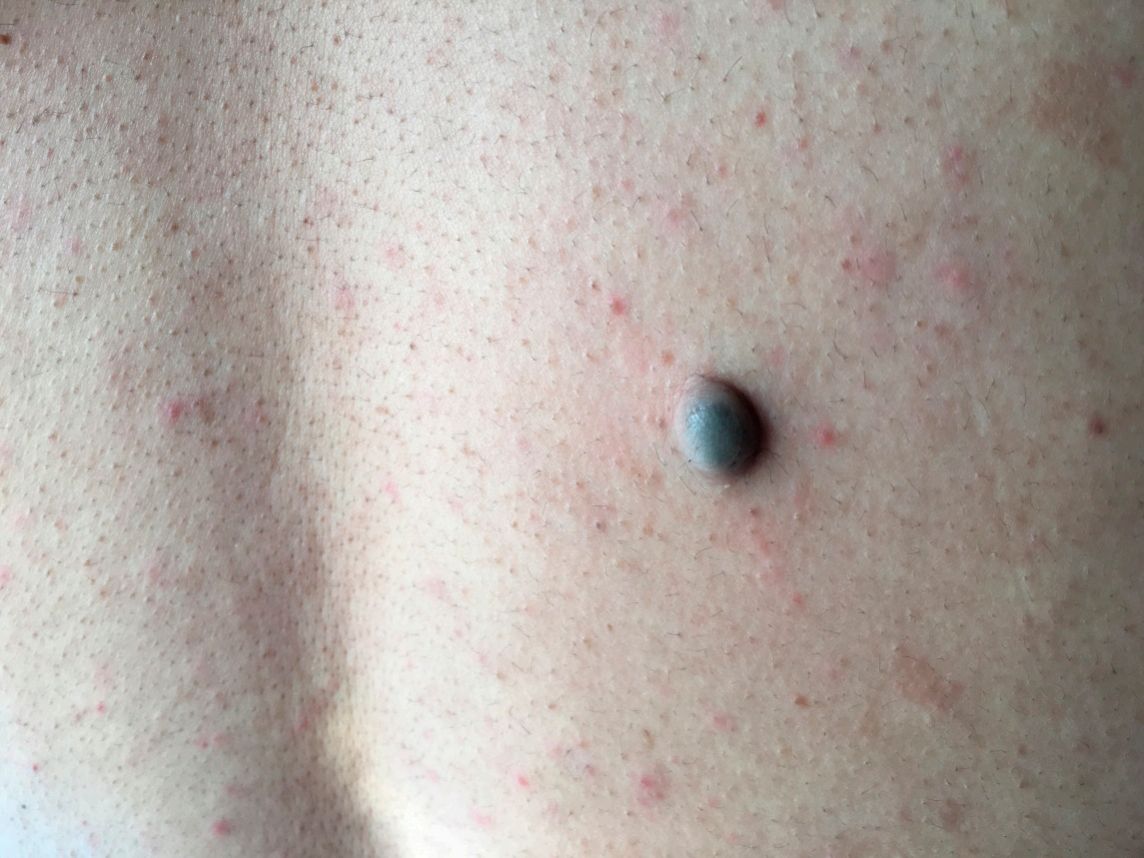
Skin lesion in the back diagnosed as pigmented schwanoma.

**Figure 3:**
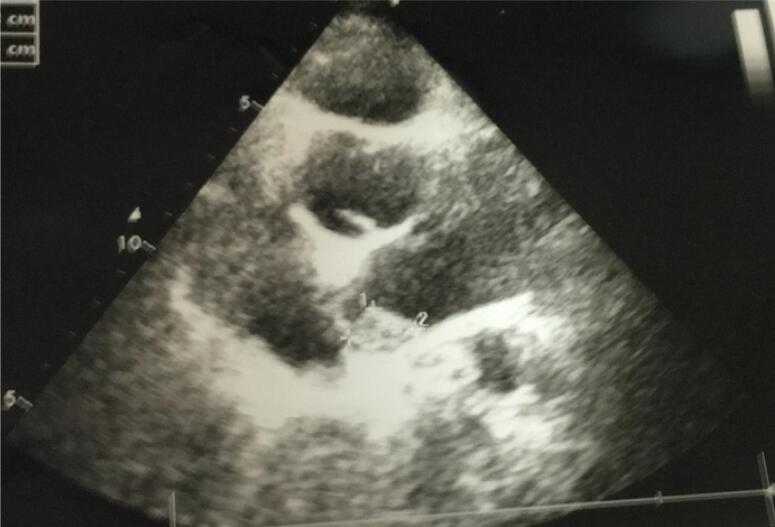
Echocardiography showed a 2.1 by 1.8 cm myxoma in base of left atrium.

**Figure 4:**
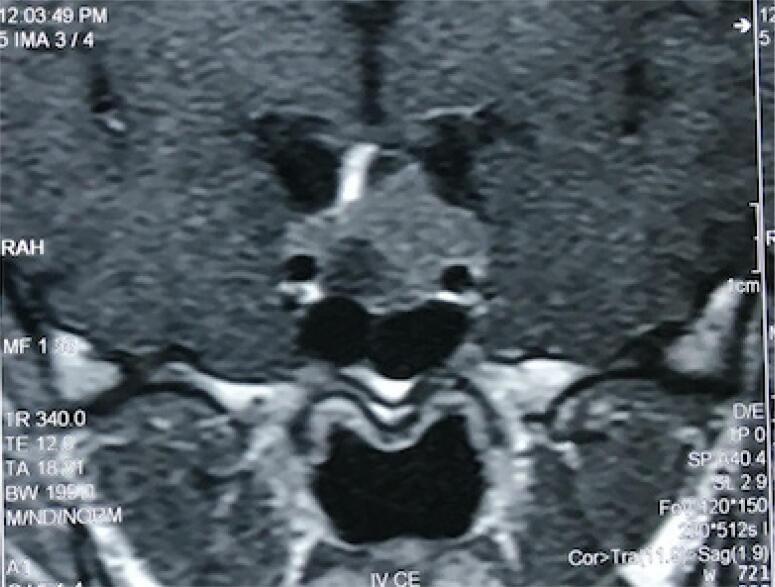
Pituitary MRI revealed a macroadenoma.

Physical examination revealed a tall young woman with a height of 186 cm and weight of 100 kg. Her face was coarse and edematous. Careful examination of the skin and mucosa revealed a pigmented macule in palpebral conjunctiva (Figure 1) and a soft tissue lesion in the back (Figure 2). Rest of physical examination was negative. Echocardiography showed a 2.1 x 1.8 cm myxoma in base of left atrium (Figure 3) and pituitary MRI revealed a macroadenoma (Figure 4). Routine laboratory evaluation was normal. Possibility of CS was proposed based on values of serum and urine cortisol; however, further evaluation of the adrenal axis was postponed because of cardiac surgery.

Cardiac surgery was uneventful and the patient recovered without complications. The soft tissue lesion of the back was also excised at same session. Histopathologic evaluation revealed atrial myxoma. The skin lesion of the patient was diagnosed as pigmented schwannoma.

Two months after surgery she was readmitted for evaluation of CS. Computerized tomographic scanning (CT) of adrenal glands revealed minimal enlargement of the glands. CT scan of abdominal and pelvic cavities for evaluation of kidneys, uterus and ovaries were negative. Ultrasound evaluation of thyroid gland and breasts were also negative. She underwent low dose and high dose dexamethasone tests; the results are shown in Table 1.

**Table 1:** Values of serum ACTH and serum and urine cortisol at baseline and after DST

Test	Baseline	LDDST	HDDST
Serum cortisol (μg/dL)	17 (14-20)	**13** (<5)	**14** (<5)
24 h UFC (μg/day)	**419** (<190)	**358** (<20)	**350** (20)
ACTH (pg/mL)	**5** (7-63)	**0.1** (<10)	**0.3** (<10)

LDDST: Low dose dexamethasone suppression test; HDDST: high dose dexamethasone suppression test. Abnormal values in bold. Reference values in brackets.

**Figure 5:**
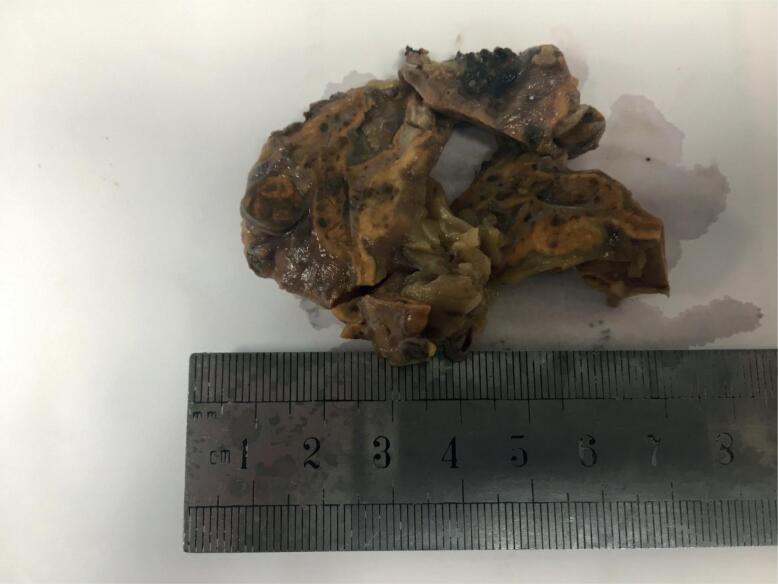
Pigmented areas could be seen dispersed in both resected adrenals.

Unsuppressed serum and urine cortisol and low plasma ACTH were in favor of diagnosis of CS and the patient was referred for laparoscopic bilateral adrenalectomy. Right adrenal was 6 cm and left adrenal was 7 cm (normal < 5 cm). Combined weight of adrenals was 25.5 grams (normal 7-10 grams). Multiple nodules could be seen overlying both adrenals. On macroscopic evaluation of the resected adrenals, pigmented areas could be seen dispersed in both adrenals (Figure 5). Histopathologic characteristics of the adrenal glands were in favor of PPNAD.

## METHODS

To evaluate the genetic characteristics of the disease, DNAs of the patient and her brother, who was clinically asymptomatic, were extracted from peripheral white blood cells and sent to the Unit on Genetics and Endocrinology, Developmental Endocrinology Branch, National Institute of Child Health for sequencing the *PRKAR1A* gene. Using genomic DNA from the submitted specimen, the coding regions and splice junctions of the requested gene were PCR amplified and capillary sequencing was performed. Bi-directional sequence was assembled, aligned to reference gene sequences based on human genome build GRCh37/UCSC hg19, and analyzed for sequence variants. This work has been conducted with the informed written consent of the patient and in accordance to the ethics committee of the Research Institute for Endocrine Sciences, Shahid Beheshti University of Medical Sciences, Tehran, Iran. All work also conforms to the provisions of the Declaration of Helsinki.

## RESULTS

The study revealed a novel mutation, c.642dupT in the patient. Gene sequencing for *PRKAR1A* in his brother failed to show any mutation. This duplication causes a frameshift starting with codon Valine 215, changes this amino acid to a cysteine residue and creates a premature stop codon at position 18 of the new reading frame, denoted p. Val215CysfsX18(V215CfsX18).

## DISCUSSION

In this paper, we present an Iranian patient with CNC who harbored a novel pathogenic mutation in *PRKAR1A* gene and presented clinically with acromegaly, recurrent atrial myxoma and CS.

Studies on patients who presented with constellation of apparently unrelated disorders such as CS, acromegaly, cardiac myxoma and mucocutaneous brown-black macules led Carney JA and his colleagues present a new form of multiple tumor syndrome (5). With increasing knowledge on this rare disorder, CNC is being diagnosed more frequently.

From clinical point of view, mucocutaneous spotty lesions are the most frequent presenting symptoms which are seen in 70%-80% of patients (1,3,6-8). The lesions are seen as brown-black macules around the lips, genitalia or in palpebral conjunctiva. Schwannoma of the skin is a rare manifestation of the disease which was seen in our patient. Atrial myxoma is seen in 20%-53% of the patients and is the most worrisome component of the syndrome that imposes major complications such as cerebral emboli, cardiac arrhythmias and congestive heart failure. It is also the leading cause of death in CNC (1,3,4,7,9,10).

CS secondary to PPNAD and acromegaly secondary to pituitary somatotroph adenoma are seen in 25%-60% (3,6-8,10) and 10%-12% of cases respectively (1,3,4,6-8). Thyroid nodules, breast ductal tumor and large cell calcifying Sertoli cell tumor (LCCSCT) of the testes are seen with lower frequency in these patients.

Diagnosis of CS secondary to PPNAD can be challenging. Occasionally, patients present with atypical CS; UFC may be normal or near-normal but cortisol diurnal rhythm is consistently abnormal (2,11). During 6-day Liddle test, there is progressive paradoxical increase in the UFC in the 6th day (2,11,12). In our patient such a pattern was not seen but there was no suppression by LDDST and HDDST and pathology clearly identified PPNAD.

Biochemical acromegaly (elevation of growth hormone and IGF-I levels) can be found in 75% of patients (10) but clinically evident acromegaly is seen less frequently in patients with CNC (13).

The mutation in our patient, duplication of T in coding sequence 642 (c.642dupT), is reported for the first time. Based on the fact that the mutation was found only in the affected member of the family, our conclusion is that the mutation is pathogenic and lack of the disease in the family of the patient points to the de novo nature of the mutation. The mutation resulted in substitution of amino acid cysteine instead of the naturally occurring valine in the peptide chain leading to a premature stop codon at position 18 (V215CfsX18). Synthesis of messenger RNA is impaired due to premature termination of the gene and the truncated mRNA is rapidly decayed without translation to protein. Failure to develop the tumor suppressor protein in those with premature stop codon renders them more susceptible to tumorigenesis. Development of multiple components of the syndrome in our patient (recurrent myxoma, acromegaly, cutaneous nevi, cutaneous schwanoma and CS) may be due to complete lack of tumor suppressive activity secondary to complete lack of the related protein.

Initially it was assumed that since mutations in CNC result in premature stop codon and subsequently non-sense mediated mRNA decay (NMD) and lack of protein production, there is no correlation between genotype and phenotype and no significant differences can be identified between CNC patients (6); however in a study on 353 patients with CNC, some genotype-phenotype correlations were reported (8). The *PRKAR1A* pathogenic mutations include missense, nonsense, frameshift, splice site mutations and sometimes large deletions which usually result in NMD but except in nonsense mutations that always result in NMD, there is also possibility of altered protein expression (1) and phenotypic diversity cannot be predicted by type of detected mutation. Overall, those mutations resulting in altered protein production are associated with higher number of CNC manifestations (8). Acromegaly, cardiac myxoma, lentigines and psammomatous melanotic schwannoma (PMS) were more often associated with exonic mutations (2,8). In 25 patients with CNC and acromegaly, one third of the mutations were in exon 3 (14). One fifth of the mutations resulted in altered protein production and in 17 patients mutations resulted in premature stop codons. In 3 patients no mutations could be defined (14).

Tumour-suppressor genes generally act in a recessive way, requiring loss of both copies to induce tumorigenesis (15); it has been proposed that tumorigenesis in CNC may be caused by second hit in different tissues (16). Unfortunately, it was not possible for us to examine surgical tissues for assessing the second hit, but at least adrenocortical tumorigenesis in CNC seems to occur apart from the second hit (15) although more studies are needed.

Management of the patient is our major concern at present. Left atrial myxoma has been successfully removed at this session, but recurrence of atrial myxoma 4 years after the first cardiac surgery is a real concern. Growth hormone hypersecretion has not yet been controlled despite 2 times of pituitary surgery and monthly injection of 20 mg sandostatin LAR. Unfortunately, pegvisomant is not available to us. Lowering serum growth hormone in this case is crucial because studies by Bandettini and cols. have shown that lowering serum GH in patients with CNC reduces the recurrence rate of cardiac myxomas (17).

In conclusion, Herein, we presented a new case of CNC who harbored a novel mutation in *PRKAR1A* gene and presented with recurrent atrial myxoma, acromegaly, CS, pigmented schwanoma of the skin and multiple cutaneous nevi.

## References

[1] Correa R, Salpea P, Stratakis CA (2015). Carney complex: an update. Eur J Endocrinol.

[2] Rothenbuhler A, Stratakis CA (2010). Clinical and molecular genetics of Carney complex. Best Pract Res Clin Endocrinol Metab.

[3] Stratakis CA (2016). Carney complex: A familial lentiginosis predisposing to a variety of tumors. Rev Endocr Metab Disord.

[4] Espiard S, Bertherat J (2013). Carney complex. Front Horm Res.

[5] Carney JA, Gordon H, Carpenter PC, Shenoy BV, Go VL (1985). The complex of myxomas, spotty pigmentation, and endocrine overactivity. Medicine (Baltimore).

[6] Stratakis CA, Kirschner LS, Carney JA (2001). Clinical and molecular features of the Carney complex: diagnostic criteria and recommendations for patient evaluation. J Clin Endocrinol Metab.

[7] Vezzosi D, Vignaux O, Dupin N, Bertherat J (2010). Carney complex: Clinical and genetic 2010 update. Ann Endocrinol (Paris).

[8] Bertherat J, Horvath A, Groussin L, Grabar S, Boikos S, Cazabat L (2009). Mutations in regulatory subunit type 1A of cyclic adenosine 5'-monophosphate-dependent protein kinase (PRKAR1A): phenotype analysis in 353 patients and 80 different genotypes. J Clin Endocrinol Metab.

[9] Siordia JA (2015). Medical and Surgical Management of Carney Complex. J Card Surg.

[10] S Boikos SA, Stratakis CA (2007). Carney complex: the first 20 years. Curr Opin Oncol.

[11] Sarlis NJ, Chrousos GP, Doppman JL, Carney JA, Stratakis CA (1997). Primary pigmented nodular adrenocortical disease: reevaluation of a patient with carney complex 27 years after unilateral adrenalectomy. J Clin Endocrinol Metab.

[12] Stratakis CA, Sarlis N, Kirschner LS, Carney JA, Doppman JL, Nieman LK (1999). Paradoxical response to dexamethasone in the diagnosis of primary pigmented nodular adrenocortical disease. Ann Intern Med.

[13] Sandrini F, Stratakis C (2003). Clinical and molecular genetics of Carney complex. Mol Genet Metab.

[14] Boikos SA, Stratakis CA (2006). Pituitary pathology in patients with Carney Complex: growth-hormone producing hyperplasia or tumors and their association with other abnormalities. Pituitary.

[15] Almeida MQ, Brito LP, Domenice S, Costa MH, Pinto EM, Osório CA (2008). [Absence of PRKAR1A loss of heterozygosity in laser-captured microdissected pigmented nodular adrenocortical tissue from a patient with Carney complex caused by the novel nonsense mutation p.Y21X]. Arq Bras Endocrinol Metabol.

[16] Pack SD, Kirschner LS, Pak E, Zhuang Z, Carney JA, Stratakis CA (2000). Genetic and histologic studies of somatomammotropic pituitary tumors in patients with the “complex of spotty skin pigmentation, myxomas, endocrine overactivity and schwannomas” (Carney complex). J Clin Endocrinol Metab.

[17] Bandettini WP, Karageorgiadis AS, Sinaii N, Rosing DR, Sachdev V, Schernthaner-Reiter MH (2016). Growth hormone and risk for cardiac tumors in Carney complex. Endocr Relat Cancer.

